# Non‐clinical considerations for supporting accelerated inclusion of pregnant women in pre‐licensure clinical trials with anti‐HIV agents

**DOI:** 10.1002/jia2.25914

**Published:** 2022-07-19

**Authors:** Rick Greupink, Hedwig van Hove, Felix Mhlanga, Peter Theunissen, Angela Colbers

**Affiliations:** ^1^ Department of Pharmacology and Toxicology Radboud Institute of Molecular Life Sciences Nijmegen Netherlands; ^2^ UZ‐UCSF Collaborative Study in Women's Health Zimbabwe Harare Zimbabwe; ^3^ CBG‐MEB Utrecht Netherlands; ^4^ Department of Pharmacy Radboud Institute for Health Sciences Nijmegen Netherlands

**Keywords:** antiretroviral pharmacology, DART, non‐clinical, pregnancy, reproduction toxicology

## Abstract

**Introduction:**

To allow the continued participation of women enrolled in pre‐licensure clinical trials who become pregnant, and to potentially enrol pregnant women in clinical trials, non‐clinical developmental and reproductive toxicology studies (DART) are essential. Generally during pharmaceutical development, DART studies are conducted late during clinical development, leading to the exclusion of pregnant women from enrolment and withdrawal of women becoming pregnant during pre‐licensure trials.

**Discussion:**

Completing all DART studies prior to or early during the conduct of phase 3 trials (i.e. earlier than current common practice) can accelerate and facilitate the inclusion of women who become pregnant during pre‐licensure trials to remain on study drug and to potentially enrol pregnant women more rapidly. Promoting complementary strategies, such as alternative combinations of DART study designs and physiologically based pharmacokinetic modelling, could better inform drug dosing and safety in pregnancy at an earlier stage in drug development. The interpretation of the results of non‐clinical DART studies is often complex. Institutional review boards/ethics committees should have access to relevant expertise for interpretation and application of results of non‐clinical developmental and reproductive toxicity studies. Clear communication and thorough understanding of non‐clinical findings and the overall benefit–risk profile of the product are critical to review protocols and determine if women who become pregnant during a clinical trial could continue on study drug and/or to enrol pregnant women in the trial. The informed consent document should be well written so that participants can make an informed decision to stay on study drug or participate in a trial during pregnancy. Ultimately, the decision to allow women who become pregnant during pre‐licensure trials to remain on study will depend on the totality of the evidence and benefit–risk considerations.

**Conclusions:**

We propose that industry completes non‐clinical reproductive toxicity studies prior to or early during the conduct of phase 3 trials in HIV drug development, especially for priority agents, and potentially uses alternative DART study design strategies to achieve this goal.

## 1 INTRODUCTION

Pregnant women, or women of childbearing potential (WOCP) without adequate contraception, have been excluded from most pre‐licensure drug trials, primarily due to concerns regarding foetal safety. However, many new drugs, particularly anti‐HIV agents to treat and prevent HIV infection, are used during pregnancy after marketing authorization despite lack of information on the adequate dose in pregnancy, placental transfer and safety for the unborn child and pregnant mother.

One of the main reasons for not including pregnant women in practice is that non‐clinical developmental and reproductive toxicology studies (DART) are generally not finalized until late phase 3. The ICH (International Council for Harmonisation of Technical Requirements for Pharmaceuticals for Human Use) guideline M3(R2) on non‐clinical safety studies for the conduct of human clinical trials and marketing authorization for pharmaceuticals describes which non‐clinical studies should have been completed prior to starting clinical trials in humans, and also which non‐clinical studies should have been completed prior to the inclusion of WOCP and pregnant women. In addition to genotoxicity (studies to test gene mutation potential) and general animal toxicology studies (including evaluation of the female and male reproductive organs), non‐clinical DART studies are recommended to support the inclusion of pregnant women in clinical trials [1]. The DART package typically consists of three different kinds of studies:

- Fertility and early embryonic development (FEED) toxicity studies, with the aim to test for adverse effects of new drugs on male and female fertility, and implantation and development of the embryo. This study is typically conducted in rodents. Treatment starts before mating and continues until after implantation and is usually of relatively short duration [2, 3].
- Embryo‐foetal development (EFD) toxicity studies, with the aim to detect adverse effects on the pregnant female and survival and development of the embryo and foetus following treatment from implantation until just prior to birth, conducted in both rodents and non‐rodents. These studies can take up to 1 year to perform and report.
- Pre and postnatal development (PPND) toxicity study, with the aim to detect adverse effects following exposure of the pregnant animal from implantation through weaning to evaluate effects on the pregnant or lactating female and development of the offspring covering two generations; it is usually conducted in rodents; however, other species can be used as appropriate [2]. The PPND study usually takes at least one and a half years to perform and report.

In general, three dose levels are tested per species in these studies. For chronic diseases, such as HIV, the duration of repeated‐dose toxicity studies is up to 6–9 months.

Current general practice is to perform the EFD and FEED studies during early phase 2 of clinical development of a new small molecule drug, so that the data are available to support the enrolment of WOCBP in phase 3 trials in all regions [2]. In the United States, WOCBP can be enrolled in phase 2 trials, in the absence of FEED/EFD studies when clinical trial participants take adequate precautions to avoid pregnancy. In regions outside of the United States, inclusion of WOCBP (up to 150) receiving investigational treatment for a relatively short duration (up to 3 months) in phase 2 trials can occur before the conduct of definitive reproduction toxicity testing. However, in this case, by default a preliminary EFD study has to be performed in two species or definitive *in vivo* testing in one of the two species can be deferred at this stage as part of an integrated testing strategy [2]. The PPND study is typically performed later in clinical development (start mid/late phase 3) to support the marketing application. Although there are examples of women staying on the (antiretroviral) study drug after becoming pregnant despite lack of PPND data, all DART data should preferably be available before a woman is allowed to remain on study drug during pregnancy, particularly for new molecular entities (e.g. drugs with new mechanism of action). Thus, PPND studies should be completed prior to allowing pregnant women to stay on study drug, to enable healthcare professionals and pregnant patients make optimal informed decisions regarding safety for mother and child.

In contrast with small molecule drugs, which follow the steps described above, for biological products, fewer non‐clinical studies are recommended to support clinical trials in pregnant women [3]. For example, to support the administration of broadly neutralizing monoclonal antibodies (bNAbs) targeting the HIV virus, conducting a tissue cross‐reactivity (TCR) study using relevant human tissues or studies using alternative protein interaction technologies (with appropriate justification) is needed to support clinical trials in pregnancy. TCR studies are *in vitro* tissue‐binding assays conducted to characterize the binding of monoclonal antibodies and related antibody‐like products to antigenic determinants in tissues. A TCR study with a panel of human tissues is a recommended component of the safety assessment package for biologicals [3]. Typically, for such products, if no specific concerns are identified in the non‐pregnant adult animal repeat‐dose toxicology and TCR studies, DART studies are not warranted, and it is possible to retain women who become pregnant on study drug in a clinical trial with bNAbs, in the absence of DART studies.

Generally, during pharmaceutical development, DART studies are conducted late during the drug development program, leading to the exclusion of pregnant women from enrolment, and withdrawal of women from pre‐licensure drug trials if they become pregnant.

To address this challenge, the IMPAACT Network and World Health Organization convened a workshop [4], which included discussions on non‐clinical studies. Building on the outcomes of the discussion, we here propose how to enable earlier generation of non‐clinical data for antiretroviral drugs to be used in pregnant women.

## 2 DISCUSSION

### 2.1 Timing of non‐clinical DART studies for new anti‐HIV agents

Although DART studies typically consist of separate studies as outlined in the introduction, alternative combinations of these study designs can also be considered, potentially shortening the duration of the conduct of the DART package and to help reduce animal use [2]. Thus, industry is encouraged to consider utilizing alternative and potentially more efficient approaches to collect DART data, for example by combining different DART studies.

Completion of FEED and EFD studies in early phase 2 and performing the PPND study prior to or early during the conduct of phase 3 would tremendously help with the benefit–risk assessment to allow women who become pregnant during clinical trial to remain on study drug (after reconsenting) and to enrol pregnant women. This strategy appears to be feasible and is in line with the ICH M3(R2) guidance on non‐clinical safety studies for the conduct of human clinical trials and marketing authorization for pharmaceuticals. PPND studies, however, are of long duration and can be very costly. Given the financial and animal welfare implications and considerations that a drug may potentially fail in phase 3, applying this accelerated approach to priority drugs [5] may be the most feasible and acceptable strategy.

In general, the timing of the conduct and availability of DART studies should be similar for HIV drugs intended for treatment or prevention [6].

### 2.2 Translation and interpretation of non‐clinical DART studies

Even when the results of non‐clinical DART studies are available during early drug development, interpretation of the results of these studies is often complex, even for the trained non‐clinical reproductive toxicologist. Multiple parameters that influence each other have to be considered when identifying hazards and/or performing risk assessment for the embryo, foetus and/or developing offspring. For the investigator/prescriber, participant (pregnant patient/WOCBP) and ethics committee members, interpretation of DART study results can, therefore, be extremely challenging. This may complicate the risk–benefit assessment when deciding on allowing the inclusion of pregnant women in clinical trials. Succinct and effective communication of non‐clinical findings and the overall benefit–risk profile of the product are critical for ethics committee members and clinicians as they review protocols to determine if the study should allow women who become pregnant during a clinical trial to continue on study drug. Also, participants require clear and easy‐to‐understand information on DART study‐generated data to make their personal decision to stay on study drug or enter a clinical trial.

The following information could help interpreting the non‐clinical DART study results: general knowledge of the product from earlier performed studies (e.g. dose–effect relationship and dose–toxicity relationship); determination of seriousness and potential reversibility of the findings; did the findings in DART studies occur at exposures relevant to humans; the influence of maternal toxicity as a potential causal factor when adverse birth outcomes are reported in DART studies; and consistency of findings across DART studies [2]. In addition, there are regional guidance documents on how to interpret these DART findings for labelling [7, 8, 9].

Developing a simplified categorization model, to guide investigators, participants and ethics committees evaluating risk and benefit, is very challenging due to the complexity of interspecies extrapolation and interpretation. For instance, when a specific developmental effect is observed at a human relevant exposure in a pharmacologically relevant non‐clinical species, the concern for human EFD with drug use is increased. Clear positive signals for embryo‐foetal toxicity (evidence of malformations or embryo‐foetal death at clinically relevant drug exposures) and clear absence of embryo‐foetal toxicity (absence of any relevant negative effect at a sufficient exposure multiple in two species) are relatively easy to identify and describe in a simplified manner. However, the main difficulty lies in how to simplify the interpretation of less severe signals of EFD toxicity (i.e. signals other than malformations/death at relevant exposures), which need broad consideration and a comprehensive explanation of the data. While animal studies are relatively good at detecting teratogenic signals, there can be poor interspecies concordance in how embryo‐foetal findings in one species will manifest in another species. Thus, signals that might be judged as less severe in the animal could, nonetheless, predict the potential for serious adverse events in humans, complicating efforts towards a simplified categorization model.

Effort should be made to improve and simplify the translation from animal to human data for stakeholders in clinical trials approval and implementation. One aspect that should be included is information on how the plasma concentrations (exposure) of the investigational product in the non‐clinical study compare to estimated human exposure in the clinical trial. Next to DART experts, both medical specialists and patient stakeholders should be involved in guiding language and ways (visuals and short movies) to inform pregnant women on the risks and benefits participating in a clinical trial. The focus should be on how to simplify the translation of DART study results for the target audience and how to improve the translation of DART study results to the human/clinical trial situation. Furthermore, an effort should be made to further educate clinical trial investigators and ethics committee members on interpretation of DART study results, and to educate DART experts in translating results into easy‐to‐interpret language.

### 2.3 Alternatives for non‐clinical DART studies

Assessment of a new pharmaceutical's potential toxicity to reproductive capacity and embryo‐foetal and postnatal development has traditionally been conducted in animal models, as described above. A number of *in vitro* methods, or *in vivo* methods using non‐mammalian organisms have been developed over the years as tools for assessing the mechanism of action of agents observed to adversely affect DART endpoints [10, 11]. *In vitro* assays and *in silico* approaches also have the potential to contribute to a weight‐of‐evidence assessment of the potential for the drug to undergo placental transfer or transfer to human milk. There has been increased interest in the possibility of using these *in vitro* models as part of the initial DART assessment. In recognition of the potential of such *in vitro* approaches, the most recent revision of the ICH S5 guidance (R3) includes a section that describes scenarios in which an appropriately qualified *in vitro* alternative assay could be used to assess embryo‐foetal toxicity in support of clinical studies in WOCBP and/or a marketing application [2]. The guidance does not describe nor endorse any specific type of alternative assay, but rather describes criteria that such assays need to meet, as well as providing reference compound data that be used in qualifying alternative assays. Currently, no alternative assays for EFD toxicity have been qualified. However, active efforts should be put in place to scan the horizon and promote research to develop and validate novel approaches in the future.

For exposure assessment, physiology‐based pharmacokinetic (PBPK) modelling is an approach that has come of age over the past decades [12]. Several mainstream PBPK modelling platforms have incorporated pregnancy modules, which incorporate physiological changes taking place throughout pregnancy. These models allow estimating changes that may take place in pharmacokinetics in pregnancy for drugs that make use of disposition pathways that have been adequately included in these models. The use of this modelling needs to be assessed on a case‐by‐case basis (as for any PBPK‐modelling approach for that matter), but to date, several examples are available in which the approach has been shown to adequately estimate maternal exposure [13, 14, 15, 16, 17, 18].

In a drug development setting, the PBPK approach may be used to predict human maternal and foetal exposure during pregnancy in an early stage of drug trials and, therefore, can help inform the probability that the dose selected for phase 2b/3 in pregnancy generates effective plasma concentrations in the mother. With respect to foetal exposure, PBPK models have also been successfully extended and parameterized with data from *ex vivo* human placenta perfusion studies or data from human cellular systems that mimic the human placental barrier *in vitro* [19, 20, 21, 22, 23]. Further standardization of the experimental approaches, consensus on how such data can be best incorporated in PBPK models and systematic evaluation of the models will help further advance this field in the coming years.

Comparing human and animal placental drug transfer may also be used to estimate how foetal exposure in non‐clinical experimental species, and any potential adverse foetal effects associated with such exposure, compare to expected human foetal exposure. This also includes PBPK‐modelling approaches. As this covers exposure assessment only, potential differences in toxicodynamics between species cannot be accounted in this manner.

Strategies, such as the implementation of alternative *in vitro* approaches and PBPK modelling, should be promoted to industry and national authorities, as supplements to the non‐clinical (FEED, EFD and PPND) studies to better inform drug dosing and safety in pregnancy in an earlier stage in drug development. However, it is expected that in the near future new *in vitro* techniques to assess reproductive toxicity will not replace animal studies, nor accelerate the collection of DART data.

## 3 CONCLUSIONS

The following key approaches should be considered to accelerate and facilitate pre‐licensure data collection to potentially support anti‐HIV agents labelling for use in pregnant women:

‐ Completing all DART studies prior to or early during the conduct of phase 3 trials for HIV drugs (when appropriate/feasible) to facilitate to allow women who become pregnant to continue study drug (after reconsenting) and to enrol pregnant women into a clinical trial.
‐ Improve and simplify the translation from animal to human data in a simplified manner for stakeholders in clinical trials.
‐ Continue to develop, improve and validate strategies that may complement the animal DART studies.

While the principles discussed in this paper are presented in the context of HIV, similar approaches could be applied to other agents, especially other anti‐infectives.

## COMPETING INTERESTS

AC has received honoraria from Merck Sharp & Dohme Corp 2021, and fee is paid to the institution. Other authors did not report competing interests.

## AUTHORS’ CONTRIBUTIONS

All authors participated in the workgroup and provided input for the paper.

## DISCLAIMER

The opinions expressed in this manuscript are those of the authors and should not be interpreted as the position of the CBG‐MEB.
